# Evaluation of an Adjustable Epidemiologic Information System

**DOI:** 10.1371/journal.pone.0014596

**Published:** 2011-01-27

**Authors:** Jiunn-Shyan Julian Wu, Fu-Yuan Shih, Chan-Hsien Chiu, Yuan-Lih Yeh, Jer-Jea Yan, Chwan-Chuen King, Mei-Shang Ho

**Affiliations:** 1 Department of Health, Taiwan Centers for Disease Control, Taipei, Taiwan, Republic of China; 2 Institute of Epidemiology, College of Public Health, National Taiwan University, Taipei, Taiwan, Republic of China; 3 Department of Emergency Medicine, National Taiwan University Hospital, Taipei, Taiwan, Republic of China; 4 Institute of Biomedical Sciences, Academia Sinica, Taipei, Taiwan, Republic of China; Tulane University, United States of America

## Abstract

**Background:**

In order to facilitate public health response and to achieve early control of infectious disease epidemics, an adjustable epidemiologic information system (AEIS) was established in the Taiwan public health network in February 2006.

**Methodology/Principal Findings:**

The performance of AEIS for the period 2006 through 2008 was evaluated based on a number of response times (RT) and the public health impact. After implementation of the system, the apparent overall shortened RT was mainly due to the shortening of personnel response time (PRT) and the time needed to draft a new questionnaire that incurred as personnel-system interface (PSI); PRT dropped from a fluctuating range of 9.8 ∼28.8 days in the first four months to <10 days in the following months and remained low till 2008 (0.88±1.52 days). The PSIs for newly emerged infectious diseases were 2.6 and 3.4 person-hours for H5N1 in 2007 and chikungunya in 2008, respectively, a much improvement from 1142.5 person-hours for SARS in 2003. The duration of each rubella epidemic cluster was evaluated as public health impact and showed a shortening trend (p = 0.019) that concurred with the shortening of PRT from 64.8±47.3 to 25.2±38.2 hours per cluster (p<0.0001).

**Conclusions/Significance:**

The first evaluation of the novel instrument AEIS that had been used to assist Taiwan's multi-level government for infectious diseases control demonstrated that it was well integrated into the existing public health infrastructure. It provided flexible tools and computer algorithms with friendly interface for timely data collection, integration, and analysis; as a result, it shortened RTs, filled in gaps of personnel lacking sufficient experiences, created a more efficient flow of response, and identified asymptomatic/mild cases early to minimize further spreading. With further development, AEIS is anticipated to be useful in the application of other acute public health events needing immediate orchestrated data collection and public health actions.

## Introduction

Effective control of infectious disease (ID) outbreaks requires a prompt public health response which depends on the ability of public health institutions to detect the initial episodes of the outbreak and the availability of tools that facilitate epidemiologic investigation and disease control. During the multi-country outbreaks of Severe Acute Respiratory Syndrome (SARS) in 2003, public health officers experienced difficulties in prompt data collection and analysis, due primarily to the lack of an efficient information system to integrate the epidemiologic, clinical and laboratory information collected during the rapidly evolving epidemic phases in different epidemic settings [1]. Anticipating that similar difficulties would continue to hamper responses to future emerging infectious diseases (EIDs), Taiwan Centers for Disease Control (Taiwan CDC) undertook a comprehensive systemic review to identify public health deficiencies [1], [2] and subsequently underwent restructuring in the post-SARS era.

### Description of the AEIS

**Figure 1:**
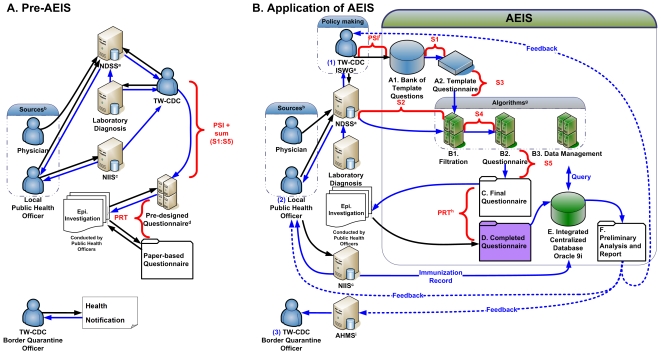
The system architecture and data flow of infectious disease case reporting in Taiwan before (Panel A) and after (Panel B) the implementation of AEIS. National Notifiable Disease Surveillance System ^a^(NDSS) receives infectious disease (ID) cases reporting from ^b^two sources: passive case finding by physicians or active case identification by public health officers. (**A**) **Prior to AEIS**: Taiwan CDC managed ID-related health information by using three information systems: NDSS, central microbiological laboratory diagnostic system, and National Immunization Information System ^c^(NIIS); for epidemic investigation, ^d^pre-designed digital questionnaires were available for 18 notifiable diseases, and paper-based questionnaires had to be generated as needed each time for all other notifiable diseases. (**B**) **After the application of AEIS**: Template questionnaires (A2) for use in investigating all 56 notifiable diseases are available in digital format. For EIDs and specific epidemiological scenarios, ^e^TW-CDC ISWG (Taiwan CDC investigation system working group) adopts the available template questions in the “question bank” (A1) to generate the template questionnaire (A2), herein, the time interval needed for this process is termed response time for generating template questionnaire (QRT), [^f^PSI (personnel-system interface)+SRT]. AEIS contains three system-operated ^g^algorithms to filter and consolidate redundant questions (B1), generate a preliminary questionnaire (B2) and re-order questions into the final version of the questionnaire (C), and integrate data management (B3) and analysis; the time period is termed system response time (SRT), 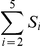. The centralized database (E) which integrated clinical, epidemiological, and laboratory data from NDSS, NIIS, and AEIS, can perform real-time analysis upon request (F). Direction of information flow is indicated with arrows in solid lines; blue indicates system-operated automatic information flow, and black indicates information flow between personnel activity (reporting and data entry) and the system. The personnel response time, ^h^PRT, is from having the final version of the questionnaire to the completion of the epidemic investigation and data entry (D), PRT. Results of the first-tier analysis will be fed back to three stake-holders (shown by dotted blue lines): (1) to Taiwan CDC ISWG for policy decision making, (2) to public health officers for fine-tuning on-site control measures, and (3) for border quarantine through the Autonomous Health Management System ^i^(AHMS).

A comprehensive web-based information system for nationwide use in all infectious disease surveillance and outbreak management, termed “adjustable epidemiologic information system” (AEIS), was established on February 13^th^, 2006. AEIS not only incorporated the existing information systems used in the national surveillance of ID (Figure 1A and 1B), i.e., the Taiwan National Notifiable Disease Surveillance System (NDSS), central microbiological laboratory diagnostic system, and National Immunization Information System (NIIS), but it also provided tools to synchronize and integrate epidemiological, laboratory and clinical information to robustly manage cases and their contacts, and to conduct data analysis under time constraints.

As shown in Figure 1A and 1B, much of the repetitive tasks executed by public health personnel in response to the occurrence of ID cases or outbreaks (Figure 1A in designing questionnaire) is replaced by computerized algorithms of the AEIS (Figure 1B steps A2 to C) as stored institutional knowledge for easy access by public health personnel. To this end, a template questionnaire for each of the 56 IDs was first installed in AEIS (Table S1). A bank of template questions (Figure 1B step A1) was compiled, and each digitalized question was conformed to standardized elements and pre-set fields which were suitable for integration and analysis by the built-in algorithms; questions related to common epidemic scenarios could be grouped into modules for easy access to be assembled into new template questionnaire for future use in EIDs.

The system flow starts with case reporting to NDSS which sends a signal for AEIS to output a template questionnaire (Figure 1B Steps A2 to C) corresponding to one of the 56 the ID to be investigated. For EIDs and specific epidemiological scenarios, a new template questionnaire (Figure 1B Step A2) could be generated by the TW-CDC investigation system working group (ISWG) by drawing from the bank of relevant questions and modules (Figure 1B Step A1). The questionnaire design would then undergo build-in algorithms to filter and consolidate redundant questions (when multiple diseases were suspected in the case) (Figure 1B Steps B1 and B2); the questions were then re-ordered into the final format of the questionnaire (Figure 1B Step C). The time interval needed for this process is termed “Response time (RT) for generating the template questionnaire” (QRT) and is made up of a personnel-system interface (PSI) and a system response time (SRT = 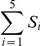) (Figure 1B Steps A1 and A2 to C), i.e., QRT = PSI+SRT. For the 56 notifiable IDs, QRT was nearly negligible as template questionnaires were built in. It is worth noting that a large portion of the personnel time that made up QRT prior to AEIS has been transformed into PSI, i.e., computer-assisted personnel time.

The questionnaire was easily accessed from the Taiwan CDC website by the field investigation team using an authorized code; the time required for the personnel activity-related interval from having the questionnaire (case report/confirmation) to the completion of epidemic investigation and data entry is designated as “Personnel RT” (PRT) (PRT in Figure 1B Steps C and D). Thus, the overall “Epidemiologic Investigation RT” (EIRT) would be PSI+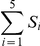
+PRT (Figure 1B: PSI, S1 to S5 and PRT), or QRT+PRT. Since this was the first evaluation of AEIS, only the overall EIRT, along with its elements, was the focus of this report.

The centralized database in AEIS (Figure 1B Step E), contains epidemiological information with integrated clinical and laboratory data from NDSS and NIIS, and has a built-in first tier data analysis algorithm to provide a real-time epidemiological report upon request (Figure 1B Step F). Results of the first-tier analysis and report can be fed back to the three stake-holders: (1) for a re-evaluation process by the Taiwan CDC ISWG advisory board for policy decisions, (2) for fine-tuning on-site control measures by public health officers, and (3) for border quarantine through the Autonomous Health Management System (AHMS). In this first evaluation of AEIS, RT of data analysis was not our focus.

### Aims of the evaluation study

AEIS is a novel information system conceptually designed to empower the public health system for a more rapid response to implement infectious disease control. The evaluation study is meant to empirically estimate the RTs of AEIS, and query specifically whether the AEIS fitted into the public health network well enough so as to shorten the PSI operating time, then we evaluated whether the shortened RTs, mostly owing to the shortened PSI, in responding to infectious disease outbreak might have a positive impact on public health (Figure S1), similar to that of a previous study in evaluating whether shortened RTs might improve the survival of patients in the settings of emergency medicine [3].

## Methods

### General design

The AEIS was evaluated for its performance based on the database compiled for the 51 acute IDs (excluding 5 chronic IDs from the 56 total notifiable IDs) during the initial period beginning on February 13^th^, 2006 through the end of 2008. In AEIS database, parameters pertaining to each individual confirmed cases included gender, birthday, nationality, county of residence, diagnosis, immunization history, occupation, travel history, medical history, history of contacts' and their immunization records, source of report, and symptoms, as well as dates of onset, reporting, case confirmation, final format questionnaire generated, first time epidemiologic investigation and data entry. Dates and methods of control measures, e.g., immunization, environmental disinfection or quarantine, were implemented also available in the database. By analyzing these parameters, all elements of RTs incurred during the flow for each outbreak response could be estimated, which was the primary goal of this study. Temporal trends of EIRTs were also studied by stratified analysis according to routes of transmission and population densities; finally the correlation between RTs and public health outcome were also studied.

In addition to instrumentation (such as AEIS), the availability of trained professionals of all levels and a harmonized vertical (central to local government) and horizontal interagency coordination are other elements in the public health infrastructure that could affect the efficiency in public health response measures (including RTs). However, these factors were not within the scope of our study as we evaluated AEIS performance focusing on how well it performed as a part of the network of the evolving public health infrastructure during the study period, rather than attempting to tease out the proportional changes of RT that was solely attributable to AEIS. Furthermore, historical EIRTs prior to the implementation of AEIS were extracted from the Taiwan CDC archived records to serve as reference measures.

### Definition of various response times

1. QRT, that is the most pertinent indicator for AEIS, is the time interval from beginning to design the questionnaire, i.e., the first EID case report triggering AEIS, to the time the template in final format is available for epidemiological investigation. QRT was the sum of two components, the personnel-system interface (PSI) and SRT (defined below).
2. SRT is included as part of QRT as the time required for the system to consolidate questions, regroup and reorganize the questions according to the logistics of obtaining information and following the algorithms for future analysis, to the final format; the precise time of execution is automatically logged by AEIS (Figure 1B: S1, S2, S3, S4, S5).
3. PRT is the time from having the final format of the questionnaire to the time of completion of the epidemic investigation and data entry; all are contributed by personnel activities and PSI during data entry (Figures 1A and 1B: PRT).
4. The “Epidemiologic Investigation RT” (EIRT), the sum of QRT (including SRT) and PRT (Figure 1B: S1 to S5 and PRT), is the interval from case reporting/confirmation to the completion of epidemic investigation and data entry.
5. “RT for case confirmation” (CRT) is the time elapsed from the date of onset to the time of case confirmation/exclusion of each EID case by laboratory diagnosis. This is largely dependent on the time required for a diagnostic test and is most pertinent for EIDs with high morbidity and mortality, as control measures include quarantine of contacts.

### Acute notifiable IDs

For the 51 acute notifiable IDs, template questionnaires were readily available via AEIS and required no modification, thus the QRT were negligible, and only the PRT was included in measuring EIRT for these diseases. We hypothesized that the implementation of AEIS might shorten the overall EIRT if the proportion of the PRT prior to AEIS which was replaced by PSI and SRT would be sufficiently shortened. Based on this premise, EIRTs were compared for time consumption prior to and after the application of AEIS (Figure 1A and 1B). Detailed information about the corresponding time frame for EIRT prior to AEIS was scattered in offices of different administrative jurisdictions; therefore, we selected shigellosis for which we could extract EIRT information from the 14 reported clusters of shigellosis outbreaks published in Taiwan Epidemiology Bulletin from 1987 to 2005 [4], [5], [6], [7], [8], [9], [10], [11], [12], [13], [14], [15], [16], [17]. The reference period prior to AEIS was divided into two parts: 1987–2003 and the post-SARS era of 2004–2005 when mass infusion of public health resources occurred; this allowed evaluation of the possible effects of other preparedness activities and resource infusion. The AEIS evaluation period was divided into 2006–2007 as the learning period and 2008 as the study end point.

For the evaluation of PRT, we conducted stratified analysis by modes of transmission [6] and by levels of population density. For the control of each ID, Taiwan CDC has proposed a guideline of time frames within which an epidemic investigation must be initiated. These were based on the routes of transmission, among other epidemic features, and different levels of urgency in responding to a specific ID (Table S1). By nature, PRT could vary according to the route of transmission. Thus, we queried whether algorithms in AEIS fitted the need of different categories of IDs. The population density was used as a surrogate for the level of urbanization and development; such a stratified analysis was based on the hypothesis that public health personnel in the remote areas, which were usually with low population density, may be less well trained and have difficulties in receiving continuing professional training and thus might have more difficulties to adapt to a new instrument (increased PSI). The population density of each administrative region (at the level of counties and cities) was grouped into high (3,000 or more people/km^2^), medium (637 to 2,999 people/km^2^), and low (636 or less people/km^2^) for analysis of their PRT.

### Application to EIDs

Based on previous experience in EIDs, QRT was mostly responsible for a lengthy EIRT. During the study period, two EIDs were added to the list of notifiable IDs, i.e. avian influenza H5N1 and chikungunya. Template questionnaires for these two EIDs were designed from scratch by reassembling questions from the bank, and this PSI interval could be calculated from the computer log. The QRT for SARS in 2003 was included for comparison (Figure 1B: PSI+PRT). For EIDs, the case definition for reporting included a category of “presumptive” case, i.e., cases fitting the clinical and/or epidemiological definition without a laboratory diagnosis, thus, CRT is an important indicator for allocation of public health resources such as implementing isolation or quarantine in the case of EIDs.

### Evaluating Asymptomatic Case Identification

All reported ID cases in the NDSS were from one of two sources: passive reporting by physicians or active case identification by epidemiologic investigation or mass screening. We hypothesized that an efficient public response would result in a reduction of case occurrence, and thus a reduction in passively reported case number, whereas the proportion of cases identified by active case finding via epidemiologic investigation, mostly likely to be asymptomatic or mild cases, might increase in proportion. We focused on diseases with higher transmissibility and different transmission routes, using shigellosis (fecal-oral transmission) and rubella (respiratory-transmission with vaccine available) diseases, to study the influence of these factors on the proportion of asymptomatic cases identified.

### Evaluating Public Health Impact

We hypothesized that a shortened RT would reduce the size and duration of each outbreak cluster so the correlations between PRT and cluster size were analyzed quantitatively. The duration of the cluster was defined as the time between the dates of onset of the index case and of the last case in the cluster. The weekly epicurve for each rubella cluster was plotted according to the week of onset of each case in each cluster in relation to the index case.

The total of 83 laboratory-confirmed rubella cases from 2007 through 2008 was analyzed using the linkage analysis algorithm of AEIS to query the spatial-temporal clustering relationship (see Text S1 in supporting information for cluster definition). The correlations among percentage of cases identified by epidemiologic investigation, PRT, and the duration of each cluster were calculated. The clusters were analyzed in chronological order based on the date of onset of the index case.

### Data analysis

The data were extracted from AEIS database and downloaded in Microsoft Excel and Access format. The statistical analyses were performed in SAS 9.1 and SPSS 14. The geographical distribution analysis was performed in Arc GIS 9.2.

The Kruskall-Wallis test was employed to test whether the median trends of PRT would be associated with different modes of transmission and areas with different levels of population density. The 95% confidence intervals for proportion of asymptomatic and mild cases identified were calculated using the Wilson score method [18] and the trends across the four stages were evaluated using the Cochran-Armitage trend test. Analysis of variance (ANOVA), *t* tests and Mann-Whitney Test were employed to test EIRT, SRT and PRT, before and after the implementation of AEIS. Associations between PRT and cluster duration were analyzed using Spearman's rho correlation coefficient (SRCC). A p-value of 0.05 was considered to be statistically significant.

## Results

### Response time

**Figure 2:**
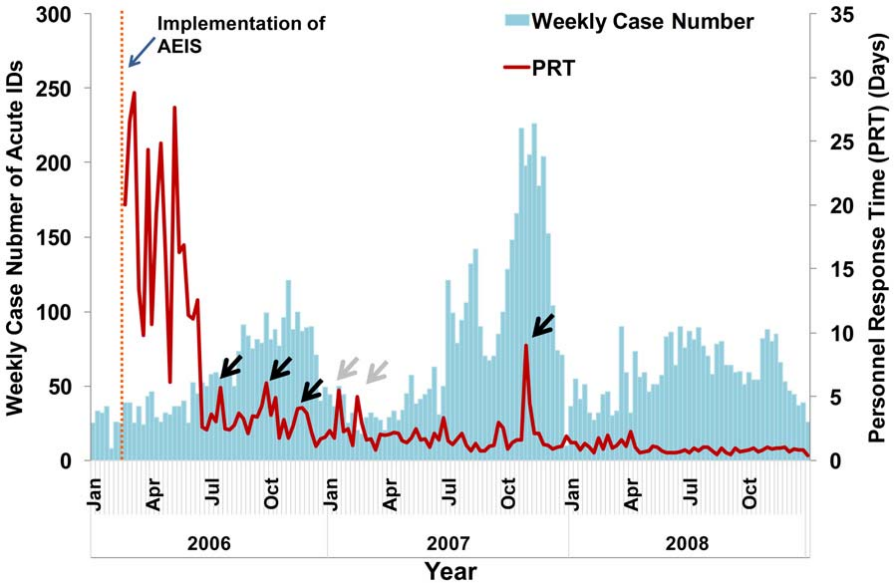
Epicurve of the 51 acute notifiable infectious diseases and its mean personnel response time by week, 2006∼2008. Left axis is the total weekly case number of the 51 notifiable acute IDs in Taiwan indicated in blue bars, January 1^st^ 2006 through December 31^st^ 2008; the right axis is the AEIS-related weekly mean personnel response times (PRT) shown in red line, February 13^th^ 2006 through December 31^st^ 2008; orange dotted line indicates the beginning of AEIS implementation. Several seemingly high mean PRT for the period coincided with the ongoing large Dengue outbreaks (marked with black arrows) occurring in 2006 through 2007; two more peaks of mean PRT (marked with grey arrows) were due to newly added one case each of HBV and HCV which were newly added to the list of IDs requiring epidemiological investigation.

For the 51 notifiable acute IDs, we first analyzed PRT based on the total 10037 ID cases registered in the AEIS database for the years 2006 (2498 cases), 2007 (4220 cases) and 2008 (3319 cases). PRT included the time needed to conduct field investigation (pure personnel time) and to enter the data (PSI). After the implementation of the AEIS on February 13^th^ 2006, the weekly mean PRT dropped precipitously from a fluctuating range between 9.8 and 28.8 days in the first four months to a range of less than 10 days in the following months (Figure 2). The decreasing trend continued with a yearly mean PRT of 6.92±22.11 days in 2006, 2.13±5.77 days in 2007, and 0.88±1.52 days in 2008. During 2006, there was no change of AEIS either the programming or computer capacity, and there was no major changes in the human resources among the local public health officials, thus the decreasing trend of PRT might be reasonably attributed to an improved PSI owing to the public health personnel having surpassed the learning period of a new instrument.

**Figure 3:**
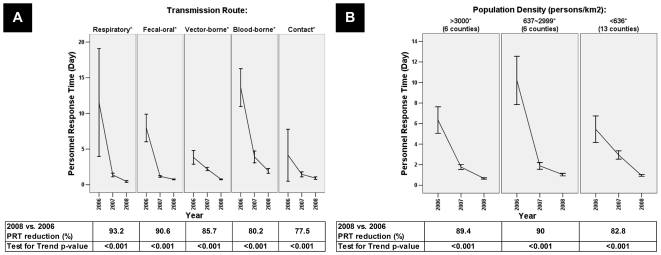
Personnel Response Time of (A) diseases category by transmission routes, and (B) areas by various population densities, February 13^th^, 2006 to December 31^st^, 2008. The personnel response time (PRT) (line) was defined as the response time from case report/confirmation to epidemiologic investigation and data entry. The horizontal bar indicates ± two standard errors (SE). An asterisk demonstrates a p-value of <0.0001. (A) PRT by routes of transmission including respiratory, fecal-oral, vector-borne, blood-borne and contact transmission. (B) PRT by areas of various population densities. The population density was classified as high (3,000 or more people/km^2^), medium (637 to 2,999 people/km^2^) and low (636 or less people/km^2^).

We then analyzed PRT by transmission route (Figure 3A), PRTs of all five groups of IDs decreased significantly over the study period without any exception, i.e., PRT reduction occurred mostly in the first two years of 2006 and 2007, and by 2008, the mean PRTs were <1 day for all but blood-borne diseases. The PRT was further evaluated for regions stratified by population density as a proxy for levels of urbanization and socioeconomic development (Figure 3B). Regardless of population density, a significant shortening of the PRT was observed over the three years following the implementation of AEIS; the overall mean PRT reduction from 2006 to 2008 was 89.4%, 90.0% and 82.8% for areas of high, medium, and low population densities, respectively (p<0.0001, Kruskall-Wallis test) (Figure 3B). The initial mean PRT of the low population density districts were similar to the high population density districts; but it is worth noting that finally it reached the comparable PRT as that of the medium level districts in 2008 (p = 0.097, Mann-Whitney Test).

Since there was no equivalent information on PRT prior to AEIS for comparison, we identified shigellosis, for which EIRT could be extracted, as a suitable disease for conducting case study to compare the overall EIRT before (reference era) and after AEIS (study era) (Table 1). The overall trend of reduction in mean EIRT was highly significant throughout the reference and study periods (p<0.001, ANOVA) (Table 1); however, the reduction of mean EIRTs between the transition of the two eras was 2.7 days. A leap higher than that of 0.6 incurred between the intra-era periods before and after AEIS.

**Table 1:** Mean Response Times (days) during the epidemiologic investigation of shigellosis cases, before and after AEIS application.

	Before AEIS Era				AEIS Era			p-value
	1987∼2003		2004∼05		2006∼07		2008	(ANOVA)
EIRT	5.1±1.4		4.5±0.5		1.8±0.1		1.2±0.1	<0.001
Reduction of EIRT*		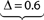		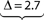		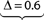		
p-value (t test)		0.43		0.01		<0.001		
SRT					0.56±0.04		0.54±0.01	0.719
PRT					1.18±0.09		0.66±0.07	<0.001

*Reduction of EIRT between the consecutive designated periods.

The mean SRT for shigellosis cases, as for all other notifiable IDs (data not shown), remained nearly unchanged during the study period, 0.56±0.04 days in 2006∼2007 and 0.54±0.01 days in 2008 (p = 0.719, ANOVA) (Table 1). In contrast, the PRT (Figure 1B, steps C to D) was greatly reduced from a mean of 1.18±0.09 days in 2006∼2007 to 0.66±0.07 days in 2008 (p<0.001, ANOVA) (Table 1). Thus, the improvement in EIRT during the first three years of AEIS was attributed primarily to the improved PRT (especially PSI).

### QRT and CRT: Application of AEIS to EIDs

A new template questionnaire would be designed as needed for each EID as it occurred. During the study period, chikungunya and H5N1 were two EIDs added to the list of notifiable diseases. The public health personnel, assisted with AEIS algorithms, selected questions from the bank of questions in modules to assemble in a template questionnaire, time required was termed PSI which was 2.6 person-hours for H5N1 and 3.4 person-hours for chikungunya. The mean QRTs were 0.68±0.01 days (range 0.67 to 0.70 days) for chikungunya and 0.107±0.416 days (range 0 to2 days) for H5N1. Both were drastically reduced from the 1142.5 person-hours required during the SARS epidemic.

The five presumptive chikungunya cases were reported between November 1 and December 31, 2008, with maximal mean PRT of 0.26±0.34 days (range 0.03 to 0.75 days). The CRT for chikungunya was >14 days, as reflected by the time required to conduct laboratory tests for specimens of convalescence period to definitively confirm or exclude chikungunya. For these reported chikungunya cases, subsequently confirmed in four (Table S3), the mean CRT of chikungunya was 11.9±10.7 days (range 2.67 to 27.74 days).

For reporting a suspected human H5N1 case, the Taiwan CDC has suggested cap of a 24-hour from reporting to the initiation of investigation using a questionnaire form modified from the WHO version [19], i.e., the QRT+SRT+PRT being one day and a 3-day cap to complete the investigation (EIRT) and the first stage control measures once the case were confirmed. While there have been no confirmed H5N1 human cases, 29 suspected H5N1 cases had been reported based on the travel history and compatible clinical syndrome (Table S4). The EIRTs of the 29 suspected H5N1 cases were all within the suggested 3 day cap. The average CRT for these 29 cases was 5.0±0.6 days with a range of 1 to 28 days as compared with a mean of 32.2±48.5 days for SARS (p<0.0001, *t* test).

### Asymptomatic and mild cases identified by epidemic investigation

We queried whether the asymptomatic and mild cases identified by epidemiologic investigation, as opposed to the passive reporting of symptomatic cases might change over time. For this analysis, we selected shigellosis and rubella as case studies, and extracted information from the reference period of January 1^st^, 2000 through February 12^th^, 2006 for comparison with the data collected from our study period.

For shigellosis cases, the average proportion of cases identified by epidemiologic investigation showed an overall decreasing trend (p = 0.0075, Cochran-Armitage test for trend) (Table 2). Of note, the total shigellosis cases also decreased from more than 200/year to 25/year in 2008. The asymptomatic fraction among these actively identified shigellosis cases was 87.5% (85.1%, 89.6%) in 2000–2003 that decreased to 20.8% (14.7%, 28.5%) in 2004–2005, and then increased to 60% (40.7%, 76.6%) in 2008 (p<0.0001) (Table 2).

**Table 2:** Proportion of shigellosis and rubella identified by epidemiologic investigation before and after the application of AEIS.

	(N)				
	Percent of cases				
	(95% confidence limits)				
ID type	Before AEIS		AEIS Application		
Case characteristics	2000∼03	2004∼05	2006∼07	2008	p-value a
Shigellosis	(818)	(130)	(145)	(25)	
Active case finding b	34.5	39.4	37.4	28.1	0.0075
	(32.9, 36.8)	(34.3, 44.8)	(35.7, 45.8)	(28.5, 52.0)	
	(716)	(27)	(44)	(15)	
Asymptomatic c	87.5	20.8	34.7	60.0	<0.0001
	(85.1, 89.6)	(14.7, 28.5)	(23.5, 38.3)	(40.7, 76.6)	
Rubella	(3)	(0)	(49)	(17)	
Active case finding	5.8	0	80.3	51.5	<0.0001
	(2.0, 15.6)		(68.7, 88.4)	(32.5, 64.8)	
	(3)		(2)	(3)	
Asymptomatic	100	-	4.1	17.6	0.1105
	(43.9, 100)		(1.1, 13.7)	(6.2, 41.0)	

a Test for trend by Cochran-Armitage: The proportion (%) that was identified by epidemiologic investigation before and after AEIS; asymptomatic cases (%) that were identified by epidemiologic investigation before and after AEIS implementation.

b Proportion of active case finding was calculated from the number of cases identified by epidemiologic investigation divided by the sum of cases identified by epidemiologic investigation and cases reported by a physician.

c Proportion of asymptomatic cases identified by epidemiologic investigation was the number of asymptomatic cases identified by epidemiologic investigation divided by the total number of cases identified by epidemiologic investigation.

The rubella cases number increased since 2007, and was found to be related to institutes with high proportion of unimmunized foreign students or workers. The proportion of rubella cases identified by epidemiologic investigation showed an increasing trend from 5.8% (2.0%, 15.6%) in the reference period to 51.5% (32.5%, 64.8%) in 2008 (p<0.0001) (Table 2). All the three rubella cases actively identified were all asymptomatic in 2000–2003, and the asymptomatic fraction increased from 4.1% (1.1%, 13.7%) in 2006–2007 to 17.6% (6.2%, 41.0%) in 2008 (p = 0.1105) (Table 2).

### Evaluating Impact of Public Health Responses

**Figure 4:**
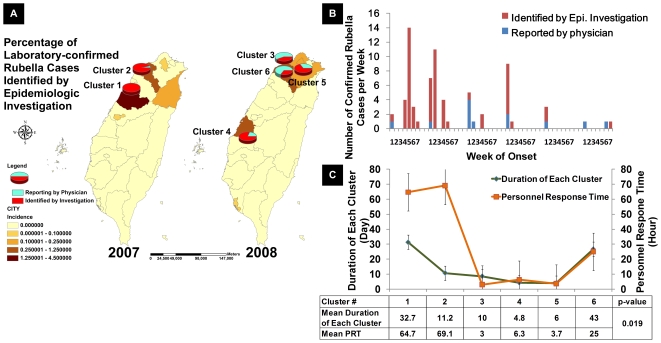
Rubella Cases in Taiwan (A) Geographical distribution of cluster cases, (B) Weekly Cases and (C) Duration of each cluster vs. mean PRT, 2007∼2008. Duration of each cluster: the time interval between the dates of onset of the index case and the last case. For an asymptomatic rubella case, the date of taking the specimen was taken as the date of onset. (A) Six spatial-temporal clusters of confirmed rubella cases were identified during 2007 and 2008 (pie charts). All clusters were located in counties with incidence higher than 0.25/10^5^ and foreigner density higher than 23/10^5^. Within each pie, cases identified by epidemiologic investigation are demonstrate in red, and cases reported by a physician in light blue. (B) The 6 rubella clusters numbered according to the time of symptom onset of the respective index case of each cluster. The weekly case numbers are presented with red bars indicating cases identified by epidemiologic investigation and light blue bars as reported by a physician according to their onset dates in relation to the onset date of the index case. The mean PRT of each cluster with standard deviation and the duration of the cluster occurrence are presented at the bottom. The date of onset for each confirmed case to the date of index onset are shown in red (cases identified by epidemiologic investigation), and light blue (cases reported by a physician) bars.

During the study period, NDSS recorded 5 epidemiologically-linked rubella clusters. This presented a good opportunity for case study to examine whether shortened PRT had any impact on public health outcomes such as the size and the duration of each cluster (Figure 4A-4C). This study extracted 83 confirmed cases reported during the study period, and an additional cluster (#6) was identified via spatial-temporal and epidemiological linkage analysis (definition of cluster refer to supporting information, Text S1). The 69 clustered cases, 83.1% of the total of 83 confirmed rubella cases, occurred in 2007 (2 clusters) and in 2008 (4 clusters) (Figure 4A). Each cluster, which was numbered according to the temporal sequence of the date of onset of the index case, consisted of 23, 23, 7, 10, 3 and 3 cases in size; the cluster size showed a decreasing trend (SRCC  = −0.912, p = 0.006). The mean PRT shortened over time with the first cluster requiring an average of 64.8±47.3 hours to the last cluster's 25.2±38.2 hours (SRCC  = −0.547, p<0.0001) (Figure 4C). Correspondingly, the general trend of the mean duration of cluster also shortened over time with the first cluster requiring 32.7±5.4 days to 4.8±2.5 days for the fourth cluster, and then 40±4.2 days for the last cluster (SRCC  = −0.086, p = 0.436). This trend of PRT shortening was concurrent with the shortened duration of the cluster (SRCC  = 0.283, p = 0.019) (Figure 4B-4C) but was not correlated with the cluster size (SRCC  = 0.647, p = 0.165).

The six rubella clusters occurred in counties with higher population densities of foreign residents [20]; the ratios of foreigners to the total number of registered residents were 0.040 in Taoyuan, 0.019 in Changhua, 0.021 in Miaoli, and 0.016 in Taipei Counties as compared to that of all other counties in Taiwan [0.016, 95% confidence interval (0.014, 0.019)] (p = 0.0125, Mann-Whitney Test). Linkage to NIIS through AEIS disclosed that all but one were expatriates who had not been immunized. The only Taiwanese case, a 21 year-old female student, was reported as having been immunized but the immunization record was untraceable.

## Discussion

To the best of our knowledge, AEIS is a unique public health instrument encompassing all functions required for ID outbreak response and management, which included questionnaire design, data collection, integration, first tier data analysis, and assistance with follow-up of cases in one package, and this is the first study to evaluate its performance. Our evaluation indicated that the public health personnel had mastered the new instrumentation of AEIS within 6 months of its implementation. During 2008, the PRT showed a pattern of diminishing drop, suggesting reaching the trough. A preliminary analysis of the mean PRT for 2009 confirmed this notion, a mean PRT of 1.22±1.18 days with maximal of 15.76 days and minimal of 0.33 days indicated that it was comparable to that of 2008. Analysis of EIDs demonstrated that AEIS could indeed fulfill the purposes of providing a flexible data collection system and rapidly tailoring it for field investigation in diverse epidemiologic scenarios.

Elements of the public health infrastructure that could affect the EIRT and efficient control measures include the availability of trained professionals at all levels, proper instruments for prompt data collection, and linkage of analysis to those making decisions in health policy. The centralized easy-to-use databank of AEIS is an important instrument that preserves continuity of the collective knowledge gathered within the public health system; in essence, it empowers and increases the proficiency of human resources. While improved EIRT within the application of AEIS was contingent on the ongoing vigilance of public health personnel in following-up and evaluating the asymptomatic cases for disease prevention and control, it should be acknowledged that AEIS probably produces an information flow system that is conducive for public health officers to remain vigilant. Another alternative explanation was that the shortened PRT was only a surrogate of an overall improvement among the elements of public health infrastructure.

### Public health RT

The bank questions and algorithms of AEIS were intended to replace the previously repetitive personnel tasks and to make it a part of the centralized easy-to-use personnel-system interface; the retrievable databank helped public health system in preserving the collective institutional knowledge. Our evaluation indicated that the shortened EIRT mainly incurred as shortened QRT, suggesting AEIS was well integrated into the existing public health systems. Our experience indicated that the mean PSI for the two EIDs were 3±0.57 person-hours each of which is much less than designing a questionnaire from scratch by person. Moreover, for H5N1, the generation of an H5N1 template questionnaire took place when H5N1 cases were happening abroad and was before the first reported suspected case occurred in Taiwan. Thus, for practical purposes, the AEIS provided a mechanism to an enhanced preparedness so that QRT for H5N1 was even shorter than other notifiable IDs. Our research was consistent with that of previous studies that found electronic device-based data collection systems saved labor and time incurred in data handling [21], [22].

### Public health impact

Our query of whether the asymptomatic and mild cases identified by epidemiologic investigation, as opposed to the passive reporting of symptomatic cases might change over time. For the case study of shigellosis and rubella demonstrated different patterns. Rubella, with vaccine available and its communicability prior to the onset of rash, finding more cases actively, especially asymptomatic ones, and implementing control measures immediately would increase the chance to blockade further transmission at the earlier epidemic waves. On the other hand, shigellosis, with no specific and effective preventive measure other than rebuilding personnel hygiene for the infected and healthy ones, the effects of identifying higher proportion of asymptomatic cases were not rewarded by an effective prevention intervention and transmission blockade. In this regard, rubella would be a better choice for public health impact of shortened PRT during epidemiologic investigation.

Outbreak clusters of rubella began in 2007 among factories and schools where high proportion of unimmunized foreigners resided (Table S2). While high correlation was found between shortened PRT and lessened size and duration of clusters, we cannot ascertain the true causal-effects of the shortened RTs and the outcomes with our available data. However, a positive health impact might be especially significant in controlling diseases that can be efficiently transmitted via asymptomatically infected individuals [23], and also diseases with a higher basic reproductive number (R_0_) [24], such as influenza, rubella and measles. Thus, one possible explanation was the use of rubella vaccine which is a specific intervention to dampen the cluster size and duration. A shortened PRT would allow earlier intervention of immunizing all contacts who showed no history of immunization (Table S2). These findings have prompted a policy of requesting foreign students and workers entering Taiwan for extended periods to receive rubella immunization prior to their entry [25].

AEIS also provided three levels of automatic electronic reminders for public health officers and related stakeholders, shortened the information cycle, and assisted in the prioritization of tasks; all can impact a shorten RT, as seen with improving the RT of the surveillance system in a manner similar to regular phone-call reminders [21]. Though the health impact seen with rubella could have been assisted by the aforementioned AEIS-related factors, however, this portion of AEIS performance was not the focus of our evaluation in this study, but is warranted in the future.

Therefore it is plausible, although further verification is warranted, that the application of AEIS in integrating laboratory information can provide a more precise estimate of R_0_ under various scenarios (e.g. schools, families, hospitals, mass-population gatherings), and possibly assess the duration and magnitude of subsequent waves during epidemics/pandemics [26], [27]. The precision of estimating R_0_ will help evaluate the effectiveness of intervention measures, such as those which occurred during the influenza A/H1N1 pandemic [28] so as to minimize the impact on health.

### Limitations and Future Prospective

In consideration of being comprehensive, this evaluation study did not cover the PSI on the semiautomatic data analysis and output; and this aspect should be the focus of the next evaluation. This study, being the first evaluation of the AEIS public health instrument, did not cover the analysis on how the one-time cost of establishing the hardware and the software programming would measure against the manpower saved in a long run, though it was evident that the information technologists within Taiwan CDC did not increase in number with the implement of AEIS. This study did not take into account the opinion of the end users of AEIS, as such a study had not been conducted formally. Future improvements in AEIS should incorporate users' acceptance, satisfaction and complaints since it remains to be seen how well AEIS may be received by public health officers. The analysis of PRT by population density did suggest a slightly lowered PRT for the districts with lower population density, it should be investigated in the future whether it was due to less trained personnel or due to the intrinsic nature of needing to travel a longer distance in serving population scattered over a larger area. In the future, it might also be worth evaluating the RT of data analysis. Finally, it would be worth evaluating the possibility of extending the application of AEIS to pharmaco-vigilance in monitoring drug and vaccine safety or in other non-ID public health emergencies.

## Supporting Information

Text S1 Supporting information for cluster definition
(0.03 MB DOC)

Figure S1 The implementation of AEIS is to have a positive impact on public health indicators, but it can not directly do so as indicated with the break of the dotted arrow. Rather, AEIS can shorten the overall EIRT by intervene a variety of RTs most notably PRT and QRT. We further analyzed the association of shortened RTs with improved public health outcome.
(0.07 MB PDF)

Table S1 List of notifiable diseases requiring epidemic investigation immediately upon reporting or after laboratory confirmation in Taiwan. ^a^AFP applied only to cases younger than 15 years of age. ^b^Syphilis and gonorrhea applied to cases younger than one year old. ^c^Dataset of HIV, AIDS and Leprosy cases have been moved to the Chronic Diseases Management System after November 1st, 2008. ^d^NDM-1-producing *Enterobacteriacea* have been classified into notifiable disease since October 9^th^, 2010.
(0.03 MB DOC)

Table S2 Epidemiologic characteristics of rubella cluster cases in Taiwan, 2007∼2008.
(0.04 MB DOC)

Table S3 Epidemiologic and clinical characteristics of reported and confirmed chikungunya cases in Taiwan, from November 1^st^ to December 31^st^, 2008. * Chikungunya has been included in notifiable diseases requiring epidemiologic investigation since November 1^st^ 2008.
(0.05 MB DOC)

Table S4 Epidemiologic and clinical characteristics of reported suspect H5N1 human cases in Taiwan, 2007∼2008. ^a^All of suspected H5N1 human cases were excluded by laboratory results. On 31^st^ May 2007, ^b^H5N1 human cases were included in notifiable disease category I, which required epidemiologic investigation as soon as cases were reported.
(0.06 MB DOC)
