# Supplementary material for: Evaluation of an Adjustable Epidemiologic Information System
Source: PLoS One. 2011 Jan 27;6(1):e14596. doi: 10.1371/journal.pone.0014596 (PMC3029279; doi:10.1371/journal.pone.0014596)
Supplement: Text S1 — Supporting information for cluster definition (0.03 MB DOC) [file pone.0014596.s001.doc]

**Supporting information:**

**Definition of cluster –** To examine the public health impact of the PRT shortening using the newly implemented AEIS, cross-period temporal and spatial analyses were performed including the laboratory-confirmed rubella cases in the datasets of AEIS from February 13^th^, 2006 to December 31^st^, 2008. Cases having epidemiologic linkage (friendship, living or working villages, and social activities) with other cases whose onset was within twice period of communicability were defined as a cluster case till the last case having linkage. Retrospectively source-tracking rubella outbreaks found were double confirmed with AEIS’ spatial-temporal and epidemiological linkage analysis algorithm, and an additional cluster (#6) was identified via this algorithm.
