# Supplementary figures and images for: Evaluation of an Adjustable Epidemiologic Information System
Source: PLoS One. 2011 Jan 27;6(1):e14596. doi: 10.1371/journal.pone.0014596 (PMC3029279; doi:10.1371/journal.pone.0014596)

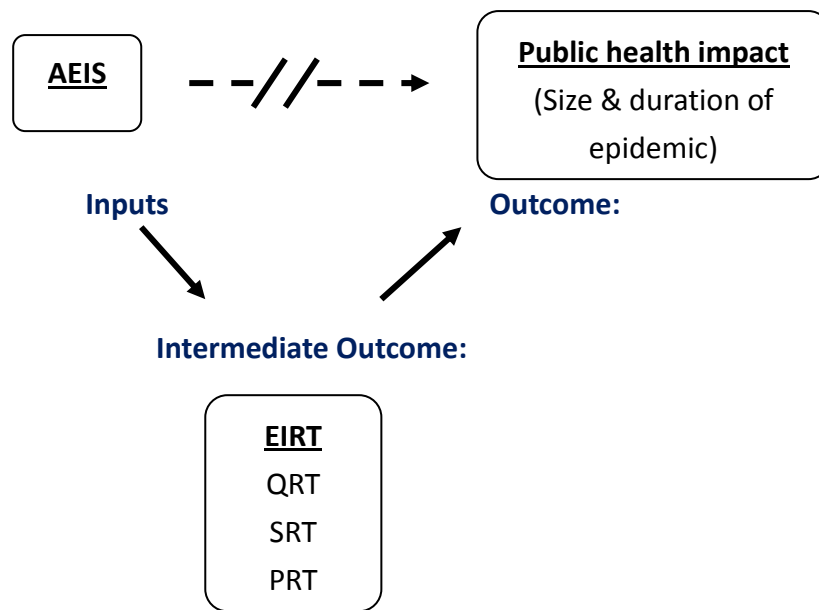

Supplement: Figure S1 — The implementation of AEIS is to have a positive impact on public health indicators, but it can not directly do so as indicated with the break of the dotted arrow. Rather, AEIS can shorten the overall EIRT by intervene a variety of RTs most notably PRT and QRT. We further analyzed the association of shortened RTs with improved public health outcome. (0.07 MB PDF) [file pone.0014596.s002.pdf]
