# Supplementary material for: Evaluation of an Adjustable Epidemiologic Information System
Source: PLoS One. 2011 Jan 27;6(1):e14596. doi: 10.1371/journal.pone.0014596 (PMC3029279; doi:10.1371/journal.pone.0014596)
Supplement: Table S1 — List of notifiable diseases requiring epidemic investigation immediately upon reporting or after laboratory confirmation in Taiwan. aAFP applied only to cases younger than 15 years of age. bSyphilis and gonorrhea applied to cases younger than one year old. cDataset of HIV, AIDS and Leprosy cases have been moved to the Chronic Diseases Management System after November 1st, 2008. dNDM-1-producing Enterobacteriacea have been classified into notifiable disease since October 9th, 2010. (0.03 MB DOC) [file pone.0014596.s003.doc]

| When to initiate | Names of diseases included in AEIS |
| --- | --- |
| As soon as a case is reported | Smallpox, Plague, Severe Acute Respiratory Syndrome , Rabies, Anthrax, Human infections with influenza A (H5N1) virus, Diphtheria, Typhoid Fever, Dengue Fever, Meningococcal Meningitis, Paratyphoid Fever, Shigellosis, Malaria, Measles, AFP^a^, Acute Hepatitis A, Enterohaemorrhagic E. coli Infection , Hantavirus Syndrome, Cholera, Rubella, West Nile Fever, Epidemic Typhus Fever, Chikungunya Fever, Pertussis, Congenital Rubella Syndrome, Acute Hepatitis B, Acute Hepatitis C, Acute Hepatitis D, Acute Hepatitis E, etc., Neonatal Tetanus, Enteroviruses Infection with Severe Complications, Syphilis^b^, Gonorrhea^b^, HIV infection^c^, AIDS^c^, Herpesvirus B Infection, Melioidosis, Botulism, Creutzfeldt-Jakob Disease, Rift Valley Fever, Marburg Haemorrhagic Fever, Yellow Fever, Ebola Haemorrhagic Fever, Lassa Fever, NDM-1–producing *Enterobacteriaceae*^d^ |
|  |  |
| As soon as the laboratory confirmation | Amoebiasis, Japanese Encephalitis, Mumps, Legionellosis, Invasive *Haemophilus Influenzae* Type b Infection, Leprosy^c^, Leptospirosis, Q Fever, Endemic Typhus Fever, Lyme Disease, Scrub Typhus, Cat-Scratch Disease |
