# Supplementary material for: Evaluation of an Adjustable Epidemiologic Information System
Source: PLoS One. 2011 Jan 27;6(1):e14596. doi: 10.1371/journal.pone.0014596 (PMC3029279; doi:10.1371/journal.pone.0014596)
Supplement: Table S2 — Epidemiologic characteristics of rubella cluster cases in Taiwan, 2007∼2008. (0.04 MB DOC) [file pone.0014596.s004.doc]

| Cluster # | Cluster size | Onset of 1^st^ Case | Onset of last case | Gender | Age | Location | % of foreigner | Constitution of contacts | Suspected source | Date of initiation Immunization |
| --- | --- | --- | --- | --- | --- | --- | --- | --- | --- | --- |
| 1 | 23 | 2007/6/1 | 2007/7/19 | 100% Male | 24.1±5.8 | Miaoli | 100%  (8.7% Thai, 91.3% Vietnamese) | There were 1500 factory workers, 680 among them were foreign workers. 31 of the foreign workers worked in the same packing department with the index case. |  | 2007/7/2 |
| 2 | 23 | 2007/6/23 | 2007/7/22 | 100% Male | 25.2±4.0 | Taoyuan | 100% Vietnamese | There were 486 foreign workers in three dormitory buildings. 371 foreign workers lived in Building B and C, and 31 of them developed symptoms. |  | 2007/7/1 |
| 3 | 7 | 2008/3/13 | 2008/4/5 | 100% Male | 19.0±0.9 | Taipei | 100% Malaysian | There were 1303 students in this university, two-thirds were foreigners. There were 83 close contacts of the index case. | Importation from returned Malaysian student | 2008/3/24 |
| 4 | 10 | 2008/4/3 | 2008/4/14 | 90% Male | 20.3±0.6 | Changhua | 90% Malaysian | There were 4293 students in this university, 69 of them were foreigners. 60 of them were close contacts | Importation from returned Malaysian student | 2008/4/9 |
| 5 | 3 | 2008/6/7 | 2008/6/13 | 0% | 21.7±2.0 | Taoyuan | 100% Vietnamese | There were 310 factory workers, 40 among them were foreign workers, and 18 of the foreign workers had close contact with the index case. |  | 2008/6/13 |
| 6 | 3 | 2008/7/2 | 2008/8/14 | 0% | 19.4±0.8 | Taoyuan | 100% Vietnamese | There were 580 factory workers, 4 of them lived in the same room. | Imported from Vietnam |  |
| Summary |  |  |  | 89.9% | 23.1±4.5 |  | 98.6% |  |  |  |
