# Supplementary material for: Evaluation of an Adjustable Epidemiologic Information System
Source: PLoS One. 2011 Jan 27;6(1):e14596. doi: 10.1371/journal.pone.0014596 (PMC3029279; doi:10.1371/journal.pone.0014596)
Supplement: Table S3 — Epidemiologic and clinical characteristics of reported and confirmed chikungunya cases in Taiwan, from November 1st to December 31st, 2008. * Chikungunya has been included in notifiable diseases requiring epidemiologic investigation since November 1st 2008. (0.05 MB DOC) [file pone.0014596.s005.doc]

| Characteristics | Positive Cases (n=4)* | Negative Cases (n=1) |
| --- | --- | --- |
| **Age (mean** ± **SD)** | 45.75±5.97 | 50 |
| **Gender** |  |  |
| Male | 4 (100%) | 1 (100%) |
| Female | 0 (0%) | 0 (0%) |
| **Symptoms** |  |  |
| Fever | 2 (50%) | 1 (50%) |
| Headache | 1 (25%) | 1 (50%) |
| Muscle pain | 1 (25%) | 0 (0%) |
| **History of Chikungunya** |  |  |
| yes | 0 (0%) | 0 (0%) |
| no | 3 (75%) | 1 (100%) |
| Uncertain | 1 (25%) | 0 (0%) |
| **Source of Infection** |  |  |
| Malaysia | 2 (50%) | 0 (0%) |
| Indonesia | 1 (25%) | 0 (0%) |
| India | 1 (25%) | 0 (0%) |
| Vietnam | 0 (0%) | 1 (100%) |
| **Purpose of Travel** |  |  |
| Business | 2 (50%) | 1 (100%) |
| Travel | 1 (25%) | 0 (0%) |
| Others | 1 (25%) | 0 (0%) |
