# Supplementary material for: Evaluation of an Adjustable Epidemiologic Information System
Source: PLoS One. 2011 Jan 27;6(1):e14596. doi: 10.1371/journal.pone.0014596 (PMC3029279; doi:10.1371/journal.pone.0014596)
Supplement: Table S4 — Epidemiologic and clinical characteristics of reported suspect H5N1 human cases in Taiwan, 2007∼2008. aAll of suspected H5N1 human cases were excluded by laboratory results. On 31st May 2007, bH5N1 human cases were included in notifiable disease category I, which required epidemiologic investigation as soon as cases were reported. (0.06 MB DOC) [file pone.0014596.s006.doc]

| Characteristics | 2007 Cases^b^ (n=19) | 2008 Cases (n=10) | 2007-2008  (N=29) |
| --- | --- | --- | --- |
| **Age (mean** ± **SD)** | 33.43±17.27 | 41.09±19.22 | 36.1±6.9 |
| **Gender** |  |  |  |
| Male | 9 (47.37%) | 4 (40%) | 13 (44.8%) |
| Female | 10 (52.63%) | 6 (60%) | 16 (55.2%) |
| **Symptom** |  |  |  |
| Fever | 14 (73.7%) | 10 (100%) | 24 (82.8%) |
| Cough | 5 (26.3%) | 7 (70%) | 12 (41.4%) |
| Acute lower respiratory diseases with unknown cause | 3 (15.8%) | 3 (30%) | 6 (20.7%) |
| Shortness of breath | 2 (10.5%) | 3 (30%) | 5 (17.2%) |
| Difficulty in breathing | 1 (5.3%) | 1 (10%) | 2 (6.9%) |
| **Risk Factors Related to Exposures** |  |  |  |
| Close contacte with H5N1 case(s) | 0 (0%) | 0 (0%) | 0 (0%) |
| Visited areas with human or avian H5N1 cases | 3 (15.8%) | 0 (0%) | 3 (10.3%) |
| Contacted wild bird(s) | 1 (5.3%) | 1 (10%) | 2 (6.9%) |
| Handled poultry | 2 (10.5%) | 1 (10%) | 3 (10.3%) |
| Ate raw avian meat | 0 (0%) | 0 (0%) | 0 (0%) |
| Close contact with other animals documented at risk to be infected with H5N1 viruses | 0 (0%) | 0 (0%) | 0 (0%) |
| Contact with possible H5N1 specimens in Lab | 0 (0%) | 0 (0%) | 0 (0%) |
| **Method of handling avians** |  |  |  |
| Feed | 1 (5.3%) | 0 (0%) | 1 (3.4%) |
| Slaughter | 1 (5.3%) | 0 (0%) | 1 (3.4%) |
| Preparation work before sales | 0 (0%) | 1 (10%) | 1 (3.4%) |
| Others | 1 (5.3%) | 0 (0%) | 1 (3.4%) |
| **Places of handling** |  |  |  |
| Indonesia | 2 (10.5%) | 0 (0%) | 2 (6.9%) |
| Viet Nam | 1 (5.3%) | 0 (0%) | 1 (3.4%) |
| **Case Ascertainment** |  |  |  |
| Meet clinical criteria and at least one epidemiologic criteria | 1 (5.3%) | 1 (10%) | 2 (6.9%) |
| Ascertain as H5N1 suspect case | 0 (0%) | 0 (0%) | 0 (0%) |
